# L-Serine Attenuates Metabolic and Behavioural Features of Diabetic Neuropathy with Dose-Dependent Central Proteomic Correlates in a Rat Model

**DOI:** 10.3390/biom16060881

**Published:** 2026-06-15

**Authors:** Menna Hamdy, Dina M. Khodeer, Mayada E. Elsakka, Ali M. Alaseem, Yasser M. Mostafa, Afaf Alharthi, Mohammad El-Nablaway, Mohamed M. Tawfik

**Affiliations:** 1 Department of Pharmacology & Toxicology, Faculty of Pharmacy, Suez Canal University, Ismailia 41522, Egypt; 2 Department of Pharmacology, College of Medicine, Imam Mohammad Ibn Saud Islamic University (IMSIU), Riyadh 13317, Saudi Arabia; 3 Department of Basic Sciences, Faculty of Physical Therapy, Horus University—Egypt, New Damietta 34517, Egypt; 4 Department of Pharmacology & Toxicology, Faculty of Pharmacy, Badr University in Cairo, Cairo 11829, Egypt; 5 Department of Clinical Laboratory Sciences, College of Applied Medical Sciences, Taif University, P.O. Box 11099, Taif 21944, Saudi Arabia; 6 Department of Basic Medical Sciences, College of Medicine, AlMaarefa University, Riyadh 13713, Saudi Arabia; 7 Department of Medical Biochemistry and Molecular Biology, Faculty of Medicine, Mansoura University, Mansoura 35516, Egypt; 8 Zoology Department, Faculty of Science, Port Said University, Port Said 42526, Egypt

**Keywords:** diabetic neuropathy, L-serine, nerve growth factor, synaptic plasticity, metabolic reprogramming, insulin resistance, proteomics, pioglitazone, LC–MS/MS, neuroprotection

## Abstract

Diabetic neuropathy (DN) is a multifactorial complication of diabetes mellitus driven by chronic hyperglycemia, insulin resistance, and disturbed metabolic homeostasis, leading to progressive injury of both the peripheral and central nervous systems. This study investigated whether L-serine supplementation could attenuate DN through dose-dependent metabolic and neuroprotective mechanisms in a high-fat diet (HFD) plus streptozotocin (STZ)-induced diabetic rat model. Male Wistar rats (n = 8 per group) were allocated to five groups: normal control (NC), diabetic control (DC), pioglitazone (PIO; 1.5 mg/kg/day), low-dose L-serine (S1; 200 mg/kg/day), and high-dose L-serine (S2; 400 mg/kg/day). After 60 days of oral gavage, behavioural testing, glucose and insulin profiling, HOMA-IR calculation, brain histopathology, nerve growth factor (NGF) immunohistochemistry, and LC–MS/MS-based proteomic analysis of cerebral tissue were performed. Diabetic rats exhibited marked hyperglycaemia (355.33 ± 4.72 mg/dL), hyperinsulinaemia, severe insulin resistance (HOMA-IR 16.8 ± 3.2; a 14-fold increase), impaired thermal nociception, motor dysfunction, and pronounced neuronal degeneration. L-serine supplementation significantly improved metabolic status: S1 reduced HOMA-IR by 77.4% and S2 by 87.5% relative to diabetic controls (*p* < 0.001). High-dose L-serine produced greater improvements in thermal sensitivity, motor coordination (rotarod latency 26.67 ± 1.52 s vs. 16.1 ± 0.85 s in DC; *p* < 0.05), and NGF expression (8.6-fold increase vs. DC). Histopathology confirmed attenuation of neuronal injury and gliosis in both treatment groups. Exploratory, group-level proteomic profiling identified dose-specific molecular signatures: S1 was predominantly associated with carbohydrate, lipid, and biosynthetic pathways, whereas S2 was associated with synaptic, neurotransmission-related, and proteostasis pathways. Within the constraints of an exploratory design—group-level pooled proteomics, analysis of cerebral rather than peripheral-nerve tissue, and only two doses—these findings indicate that L-serine attenuates the metabolic and behavioural features of experimental diabetic neuropathy and generates the testable hypothesis of dose-dependent neuro-metabolic remodelling. The proteomic signatures are hypothesis-generating and require orthogonal validation before any mechanistic or translational inference can be drawn.

## 1. Introduction

Diabetes mellitus is a chronic metabolic disorder characterized by persistent hyperglycemia caused by impaired insulin secretion, insulin action, or both, ultimately leading to progressive multiorgan dysfunction [1]. Long-standing diabetes is associated with a spectrum of microvascular complications—including retinopathy, nephropathy, and neuropathy—that substantially increase morbidity and mortality [2,3]. Among these, diabetic neuropathy (DN) is one of the most prevalent and debilitating complications, affecting up to half of patients with poorly controlled or long-standing diabetes [4,5,6]. DN manifests through progressive damage to peripheral and central neural pathways, resulting in sensory loss, neuropathic pain, motor dysfunction, and increased risk of foot ulceration and amputation [7,8,9]. Structurally, DN is characterized by axonal degeneration, demyelination, Wallerian degeneration, and impaired nerve regeneration [10,11]. Despite its substantial clinical burden, DN lacks effective disease-modifying therapies; current treatments are largely symptomatic, addressing pain rather than the underlying mechanisms of neurodegeneration [12,13,14].

The pathogenesis of DN is multifactorial, involving metabolic disturbances, mitochondrial dysfunction, inflammation, oxidative stress, and impaired neurotrophic support [4,15]. Sustained hyperglycemia triggers advanced glycation end-product formation, lipid peroxidation, microvascular ischemia, and activation of polyol and hexosamine pathways, all contributing to neuronal injury [6,16]. Metabolic memory further perpetuates neurodegeneration even after glycemic control is improved [3]. Although significant advances have been made in understanding DN mechanisms, major gaps remain in clarifying how metabolic dysregulation interacts with neuronal signalling pathways. This limits the development of targeted interventions aimed at restoring neuronal homeostasis rather than merely alleviating symptoms.

Current therapeutic strategies with disease-modifying potential—such as benfotiamine, alpha-lipoic acid, antioxidants, and agents targeting mitochondrial function—provide limited benefit and inconsistent efficacy [14,17]. Pioglitazone, a PPAR-γ agonist with anti-inflammatory and insulin-sensitizing properties, has shown partial neuroprotective effects by enhancing glucose metabolism, reducing oxidative stress, and modulating inflammatory pathways [12]. However, the therapeutic response remains variable, and the molecular mechanisms underlying its neuroprotective actions are incompletely defined. Thus, there is a significant need for alternative or adjunctive therapies that target both metabolic dysfunction and neuronal repair.

L-serine has recently emerged as a promising candidate in this context. A non-essential yet conditionally indispensable amino acid, L-serine plays critical roles in neuronal development, sphingolipid synthesis, mitochondrial function, and one-carbon metabolism [18]. Altered serine metabolism has been implicated in type 1, type 2, and gestational diabetes [19], and supplementation has been shown to improve insulin secretion, glucose homeostasis, and mitochondrial efficiency [18,20]. L-serine is also a precursor for neuroactive molecules including D-serine, glycine, and phosphatidylserine, which support synaptic stability, neurotransmission, and neuronal survival [21]. Preclinical evidence suggests that L-serine reduces neurotoxic deoxysphingolipids, attenuates neuronal damage, and slows neuropathy progression [20,22]. In diabetic models, L-serine supplementation improved nerve conduction and reduced neuropathological changes, and its long-term administration is considered safe [22]. Despite these advances, the precise dose-dependent molecular mechanisms through which L-serine exerts neuroprotective effects remain unknown.

Proteomics provides a powerful approach to elucidate the complex molecular events underlying DN. High-throughput LC–MS/MS-based proteomics enables comprehensive profiling of protein expression patterns, signalling pathways, and post-translational modifications that drive neuropathology [23]. Proteomic studies in diabetic nerve tissues have identified disruptions in energy metabolism, mitochondrial function, oxidative stress responses, and cytoskeletal organization [24,25]. However, no studies to date have characterized the global proteomic alterations in brain tissue following L-serine therapy in DN, nor have they investigated whether different doses of L-serine produce distinct molecular signatures. Understanding dose-specific proteomic responses could clarify the mechanistic basis of L-serine’s therapeutic action and identify biomarkers of treatment efficacy.

Given the lack of disease-modifying treatments for DN, the growing evidence supporting L-serine’s metabolic and neuroprotective roles, and the absence of mechanistic proteomic studies evaluating its dose-dependent effects, there remains a critical knowledge gap. Most available treatments target either metabolic dysfunction or neuroprotection, but few simultaneously address both aspects. Furthermore, DN affects both peripheral and central neural pathways, yet the molecular consequences of diabetes on brain tissue—and its response to therapeutic interventions—are underexplored.

Therefore, this study sought to investigate the functional, metabolic, histopathological, and proteomic effects of low-dose and high-dose L-serine supplementation in a rat model of streptozotocin-induced diabetic neuropathy. By integrating behavioural assessments, metabolic measurements, immunohistochemistry, histological evaluations, and LC–MS/MS proteomics, this work aims to delineate how L-serine modulates pain sensitivity, motor function, insulin resistance, neuronal integrity, and protein expression networks in diabetic brain tissue. Additionally, the therapeutic profile of L-serine was compared with pioglitazone to contextualize its efficacy relative to an established insulin-sensitizing agent.

We hypothesized that L-serine produces dose-specific neuroprotective effects in diabetic neuropathy, mediated by distinct proteomic signatures involving metabolic reprogramming, synaptic plasticity, and neurotrophin-related pathways. Specifically, we postulated that low-dose L-serine would primarily restore metabolic and biosynthetic processes, whereas high-dose L-serine would enhance synaptic, neurotransmission-related, and proteostasis pathways. By testing these hypotheses, this study provides novel mechanistic insights into L-serine’s therapeutic potential and establishes a foundation for its future clinical translation in the management of diabetic neuropathy.

## 2. Materials and Methods

### 2.1. Experimental Animals and Housing

A total of 40 adult male Wistar rats, with initial body weights ranging from 225 to 290 g, were obtained from the animal research facility of the Faculty of Pharmacy, Suez Canal University, Ismailia, Egypt. All animals were housed under standardized environmental conditions: room temperature maintained at 22 ± 2 °C, relative humidity of 55 ± 10%, and a 12:12 h light–dark cycle (lights on at 07:00). Rats had free access to standard laboratory chow and tap water throughout the acclimation period. Following arrival, animals were allowed a one-week acclimatization period to adapt to the housing conditions and to minimize stress-related physiological variations before any experimental manipulation. All animals were of the Wistar strain (an outbred albino rat strain); this is stated for clarity to prevent any confusion with other albino strains such as Sprague–Dawley. Only male rats were used to avoid the confounding effects of cyclical 17β-oestradiol fluctuations on glucose homeostasis, insulin sensitivity, and nociceptive thresholds, all of which are primary endpoints in this study; the limitations of a single-sex design are addressed in the Section Limitations of the Study. No mortality occurred in any group across the entire experimental period.

### 2.2. Induction of Type 2 Diabetic Neuropathy Model

Type 2 diabetes mellitus was induced using a combination of high-fat diet (HFD) feeding followed by a single low-dose streptozotocin (STZ) injection, a well-established model that recapitulates the metabolic and neuropathic features of human type 2 diabetes. For the initial six weeks (weeks 1–6 of the experimental timeline), all rats designated for diabetes induction were fed a high-fat diet consisting of 60% kcal from fat (Research Diets, Inc., New Brunswick, NJ, USA). Rats in the normal control group received a standard chow diet containing approximately 10% fat throughout the study period.

At the end of week 6, after an overnight fast (12 h), diabetic groups received a single intraperitoneal (IP) injection of STZ at a dose of 30 mg/kg body weight. STZ (Sigma-Aldrich, St. Louis, MO, USA) was freshly dissolved in ice-cold 0.1 M citrate buffer (pH 4.5) immediately before injection. The 30 mg/kg dose was selected to induce partial beta-cell dysfunction while preserving residual insulin secretory capacity, thereby mimicking the insulin-resistant, hyperinsulinemic phase of early type 2 diabetes.

Seventy-two hours after STZ injection, fasting blood glucose (FBG) levels were measured from tail vein blood samples using a calibrated glucometer (OneTouch Ultra 2, LifeScan, Inc., Milpitas, CA, USA). Rats with FBG ≥ 250 mg/dL were considered diabetic and included in the study. This threshold was chosen to ensure the presence of sustained hyperglycemia consistent with established diabetic neuropathy models.

### 2.3. Experimental Groups and Sample Size

Diabetic rats were randomly stratified into four experimental groups (n = 8 per group), with an additional normal control group (n = 8), resulting in five groups total: Normal control (NC): non-diabetic rats fed standard chow and receiving vehicle (sterile normal saline, oral gavage daily). Diabetic control (DC): HFD + STZ-induced diabetic rats receiving vehicle (sterile normal saline, oral gavage daily). Pioglitazone-treated diabetic group (PIO): diabetic rats treated with pioglitazone (1.5 mg/kg/day, oral gavage). Low-dose L-serine group (S1): diabetic rats treated with L-serine (200 mg/kg/day, oral gavage). High-dose L-serine group (S2): diabetic rats treated with L-serine (400 mg/kg/day, oral gavage). Sample size (n = 8 per group) was estimated a priori using G*Power 3.1 for a one-way ANOVA across five groups, with α = 0.05, power (1 − β) = 0.80, and an anticipated large effect size (f = 0.50) based on pilot hot-plate latency data. The calculation indicated a minimum of six animals per group; we enrolled eight to accommodate potential exclusions while maintaining statistical power.

### 2.4. Drug Preparation and Administration

Pioglitazone: Pioglitazone hydrochloride (Sigma-Aldrich, St. Louis, MO, USA) was suspended in sterile normal saline and administered once daily by oral gastric gavage at a dose of 1.5 mg/kg body weight in the same gavage volume (10 mL/kg) used for the L-serine arms. This dose corresponds—via allometric body-surface-area conversion—to a human-equivalent dose of approximately 0.24 mg/kg, or about 17 mg/day for a 70 kg adult, which falls within the clinically prescribed range of 15–30 mg/day, and has been used as the active comparator in published rodent studies of pioglitazone neuroprotection in diabetic and neurodegenerative models.

L-serine: L-serine (purity ≥ 99%; Sigma-Aldrich, St. Louis, MO, USA) was dissolved in sterile normal saline at working stock concentrations of 20 mg/mL (S1) and 40 mg/mL (S2). Low-dose animals (S1) received 1 mL of the 20 mg/mL stock per 100 g of body weight, delivering 200 mg of L-serine per kg per day; high-dose animals (S2) received 1 mL of the 40 mg/mL stock per 100 g of body weight, delivering 400 mg/kg per day. Both arms were dosed once daily by oral gastric gavage at a volume of 10 mL per kg body weight (i.e., 1 mL per 100 g). Stock concentrations were re-adjusted weekly to the most recent body-weight measurement to maintain dose accuracy across the 60-day treatment period. The selected doses fall within the published neuroprotective range for L-serine in rodents. Body weights were recorded weekly throughout the study to enable accurate dose adjustment; aggregated body-weight curves are not reported as a primary endpoint of this preclinical study, which focused on metabolic, behavioural, histological, immunohistochemical, and proteomic outcomes.

Treatment duration: All treatments (pioglitazone, S1, and S2) were initiated immediately after confirmation of diabetes (72 h post-STZ) and continued for 60 consecutive days. The vehicle control groups (NC and DC) received an equivalent volume of sterile normal saline by oral gavage on the same schedule.

### 2.5. Rationale for Brain Tissue Analysis in Diabetic Neuropathy

Although diabetic neuropathy is classically characterized by peripheral nerve damage, accumulating evidence indicates that chronic hyperglycemia and insulin resistance also induce significant central nervous system alterations, including neuroinflammation, synaptic dysfunction, and neuronal degeneration in brain regions involved in sensory processing, motor coordination, and cognition. Therefore, brain tissue was selected as the primary substrate for histopathological, immunohistochemical, and proteomic analyses to investigate the central neuroprotective effects of L-serine. Peripheral neuropathy was functionally confirmed by behavioural testing (tail-immersion, hot-plate, and rotarod tests) performed prior to tissue collection.

### 2.6. Behavioural Assessments

All behavioural tests were conducted during the final week of the treatment period (days 56–60 post-STZ) in a quiet, temperature-controlled room (22 ± 1 °C) under dim lighting conditions. Animals were habituated to the testing environment for 30 min before each test. All tests were performed between 09:00 and 14:00 to minimize circadian variation.

#### 2.6.1. Hot Plate Test

Thermal nociception was evaluated using a hot-plate apparatus (Model LE 7406, LSI LETICA, Rome, Italy). The apparatus consisted of a transparent glass cylinder (20 cm diameter × 25 cm height) with a digitally controlled heated surface. The plate temperature was maintained at 55 ± 0.5 °C. Each rat was placed individually onto the heated surface, and the latency to the first sign of hind paw licking, flicking, or jumping was recorded in seconds. A cut-off time of 45 s was imposed to prevent tissue injury. The surface was thoroughly cleaned with 10% ethanol solution and dried between each trial. Each rat was tested three times with a 5 min inter-trial interval, and the mean latency was calculated for analysis.

#### 2.6.2. Tail-Immersion Test

Thermal nociception was further assessed using the tail-immersion test. Rats were gently restrained in a clear acrylic chamber placed on a glass surface maintained at 25 °C. After a 30 min acclimation period, the distal third of the tail was immersed in a water bath maintained at 52 ± 0.5 °C. The latency to a rapid tail flick or withdrawal was recorded using a digital stopwatch. A cut-off time of 10 s was applied to avoid thermal injury. The test was repeated three times at 5 min intervals, and the average withdrawal latency was calculated for each rat.

#### 2.6.3. Rotarod Test

Motor coordination and balance were evaluated using a rotarod apparatus (Ugo Basile, Gemonio, Italy) consisting of a rotating rod (3 cm diameter) with a textured surface to prevent slipping. Rats were trained for two consecutive days prior to testing (three trials per day, 15 rpm, 120 s maximum). On the test day, rats were placed on the rod, which rotated at a constant speed of 15 rpm. The latency to fall from the rod was recorded, with a maximum cut-off time of 300 s. Each rat underwent three trials with a 10 min inter-trial rest period, and the mean latency was calculated for statistical analysis.

### 2.7. Metabolic Assessments

#### 2.7.1. Fasting Blood Glucose

After a 12 h overnight fast, blood samples were collected from the tail vein, and glucose levels were measured using a calibrated blood glucose monitoring system (OneTouch Ultra 2, LifeScan, Inc., Milpitas, CA, USA). Results were recorded in milligrams per deciliter (mg/dL).

#### 2.7.2. Serum Insulin

Blood samples were collected after a 6 h fast, allowed to clot at room temperature for 30 min, and centrifuged at 3000 rpm for 10 min at 4 °C. Serum was separated and stored at −80 °C until analysis. Serum insulin levels were quantified using a commercially available enzyme-linked immunosorbent assay (ELISA) kit (Millipore, Billerica, MA, USA) according to the manufacturer’s protocol. Briefly, serum samples were diluted 1:100 in assay buffer and added to wells pre-coated with anti-insulin antibody. After the incubation and washing steps, biotinylated detection antibody and streptavidin-horseradish peroxidase (HRP) conjugate were added. The reaction was developed with tetramethylbenzidine (TMB) substrate, stopped with acidic solution, and absorbance was read at 450 nm using a microplate reader (BioTek Instruments, Winooski, VT, USA). Insulin concentrations were calculated from a standard curve generated using known concentrations of rat insulin.

#### 2.7.3. HOMA-IR Calculation

Insulin resistance was estimated using the Homeostasis Model Assessment of Insulin Resistance (HOMA-IR) according to the standard formula:

HOMA-IR = [Fasting glucose (mg/dL) × Fasting insulin (µU/mL)]/405

This validated surrogate marker provides an indirect measure of whole-body insulin sensitivity based on steady-state glucose and insulin concentrations.

### 2.8. Tissue Collection and Processing

At the end of the 60-day treatment period, rats were euthanized by cervical dislocation under deep anesthesia. Brains were rapidly excised, washed in ice-cold normal saline (0.9% NaCl) to remove residual blood, and divided midsagittally. The left hemisphere was fixed in 10% neutral-buffered formalin for histopathological and immunohistochemical analyses. The right hemisphere was flash-frozen in liquid nitrogen and stored at −80 °C for subsequent proteomic analysis.

### 2.9. Histopathological Evaluation

Fixed brain tissues were processed through graded ethanol series, cleared in xylene, and embedded in paraffin wax. Serial coronal sections (3 µm thickness) were cut using a rotary microtome (Leica RM2255, Leica Biosystems, Nussloch, Germany) and mounted on glass slides. Sections were deparaffinized, rehydrated, and stained with hematoxylin and eosin (H&E) following standard protocols. Stained sections were examined under a light microscope (Olympus BX53, Olympus Corporation, Tokyo, Japan) by an independent pathologist blinded to group allocation. Histopathological features evaluated included neuronal morphology, perineuronal edema, the presence of degenerating (red) neurons, gliosis, and any other pathological alterations.

### 2.10. Immunohistochemistry for Nerve Growth Factor (NGF)

Brain sections (4 µm thickness) from paraffin-embedded blocks were mounted on poly-L-lysine-coated slides. Sections were deparaffinized, rehydrated, and subjected to antigen retrieval by heating in 10 mM sodium citrate buffer (pH 6.0) for 15 min in a microwave oven. Endogenous peroxidase activity was quenched with 3% hydrogen peroxide in methanol for 10 min. Non-specific binding was blocked with 5% normal goat serum in phosphate-buffered saline (PBS) for 1 h at room temperature.

Sections were then incubated overnight at 4 °C with a rabbit polyclonal primary antibody against NGF (Lot: bsm-52362R, Bioss Inc., Woburn, MA, USA) diluted 1:200 in blocking buffer. After washing with PBS, sections were incubated with horseradish peroxidase (HRP)-conjugated goat anti-rabbit secondary antibody (Bioss Inc.) for 1 h at room temperature. Immunoreactivity was visualized using 3,3′-diaminobenzidine (DAB) as the chromogen, and sections were lightly counterstained with hematoxylin for 30 s, dehydrated, and mounted with coverslips.

Semi-quantitative analysis: NGF immunoreactivity was assessed using the Allred scoring system, which combines the percentage of positive cells (score 0–5) and staining intensity (score 0–3). The percentage scores were assigned as follows: 0 = no positive cells, 1 = <10%, 2 = 10–25%, 3 = 26–50%, 4 = 51–75%, 5 = >75%. Intensity scores were: 0 = no staining, 1 = weak, 2 = moderate, 3 = strong. The final Allred score was the sum of these two scores (range 0–8). Additionally, the percentage area of NGF immunostaining was quantified in three randomly selected high-power fields (HPF, 400× magnification) per section using ImageJ software (version 1.53, National Institutes of Health, Bethesda, MD, USA). All analyses were performed by an investigator blinded to the experimental groups. NGF immunoreactivity was assessed in three predefined brain regions per animal—hippocampus (CA1 and CA3 pyramidal layers), parietal cortex (layers II–V), and brainstem nuclei—using three non-adjacent coronal sections per region per animal (≥60 μm apart), with three randomly selected high-power fields per section.

### 2.11. Proteomic Analysis by Label-Free LC-MS/MS with NSAF Quantification

Proteomic analysis of brain tissue was performed at the Proteomics and Metabolomics Unit, Department of Basic Research, Children’s Cancer Hospital Egypt 57357 (CCHE-57357), Cairo, Egypt—directed by Dr Sameh Magdeldin—using the unit’s standard label-free LC-MS/MS workflow with quantification by Normalized Spectral Abundance Factor (NSAF) [26,27,28,29]. Tissue samples were submitted to the unit in November 2021 and analyzed on the dates encoded in the raw data filenames (10 and 22 November 2021); the unmodified raw facility output is provided as Supplementary Table S1. The protocol below describes the procedures performed at the unit and the post-acquisition analysis performed in our laboratory; the same NSAF workflow has been described in detail in recent peer-reviewed publications from the unit [29].

Protein extraction and digestion: For each group (NC, DC, PIO, S1, S2), brain hemispheres from individual animals (n = 8 per group) were processed and equal-mass aliquots were combined into a single representative pooled sample per group prior to LC-MS/MS analysis. Frozen tissue was ground to a fine powder under liquid nitrogen and homogenized in lysis buffer containing 8 M urea, 50 mM triethylammonium bicarbonate (TEAB, pH 8.0), and a protease inhibitor cocktail. Homogenates were sonicated on ice and centrifuged at 12,000× *g* for 20 min at 4 °C. The supernatant was collected, and protein concentration was determined by the bicinchoninic acid (BCA) assay. From each pooled sample, 200 μg of protein was reduced with 10 mM dithiothreitol (DTT) at 56 °C for 1 h, alkylated with 20 mM iodoacetamide in the dark at room temperature for 45 min, and digested with trypsin (1:50 enzyme-to-protein ratio) overnight at 37 °C. Pooling at the group level was chosen at the facility to enable a discovery-phase survey of the brain proteome across all five experimental groups within a single analytical batch; we acknowledge that this design averages out inter-animal variability at the MS run level, and we address the inferential consequences in the Section Limitations of the Study and in the orthogonal-validation plan for follow-up work.

Peptide clean-up: Tryptic digests were desalted on C18 solid-phase-extraction cartridges, dried under vacuum centrifugation, and reconstituted in 0.1% formic acid for direct LC-MS/MS injection. No isobaric chemical labelling was used; quantification was achieved post-acquisition by the label-free NSAF strategy described below [26,27].

Peptide fractionation: Peptides were fractionated by high-pH reversed-phase liquid chromatography on a Shimadzu LC-20AD HPLC system (Kyoto, Japan) using a Gemini-NX C18 column (3 μm, 2 × 150 mm; Phenomenex, Torrance, CA, USA) with a linear gradient of 5–80% buffer B (20 mM ammonium formate in 80% acetonitrile, pH 10) over 60 min at 200 μL/min. Elution was monitored at 214 and 280 nm. Fractions were collected every minute and pooled into 24 final fractions on the basis of chromatographic peak intensity, then concentrated by vacuum centrifugation.

LC-MS/MS analysis: Dried fractions were reconstituted in 0.1% trifluoroacetic acid (TFA) and analyzed on an Orbitrap Q Exactive HF mass spectrometer (Thermo Fisher Scientific, Waltham, MA, USA) coupled with a Thermo Dionex Ultimate 3000 RSLC nano-system. Peptides were loaded onto a PepMap C18 trapping column (2 μm, 75 μm × 20 mm) and separated on a PepMap C18 reversed-phase analytical column (2 μm, 75 μm × 150 mm, 100 Å) at a flow rate of 300 nL/min. The mobile phases consisted of buffer A (0.1% formic acid in water) and buffer B (80% acetonitrile, 0.1% formic acid), with a linear gradient of 4–90% buffer B over 65 min.

Mass spectrometry data were acquired in data-dependent acquisition (DDA) mode with a top-20 method. Full MS scans were acquired at a resolution of 60,000 (*m*/*z* 350–1800), and higher-energy collisional dissociation (HCD) fragmentation was performed on the top 20 precursor ions with a normalized collision energy of 35%. The automatic gain control (AGC) target was set to 3 × 10^6^, with a maximum injection time of 50 ms. Dynamic exclusion was set to 40 s.

Protein identification and label-free quantification: Raw MS data were processed using Proteome Discoverer (version 2.5; Thermo Fisher Scientific) with the SEQUEST HT search engine against the UniProt Rattus norvegicus reference proteome (downloaded February 2024). Search parameters: trypsin with up to two missed cleavages; precursor mass tolerance 10 ppm; fragment mass tolerance 0.02 Da; carbamidomethylation of cysteine as a fixed modification; oxidation of methionine; and acetylation of protein N-termini as variable modifications. Peptide spectral matches were filtered to a false discovery rate ≤ 1% using the Percolator algorithm. Protein-level abundance was quantified by Normalized Spectral Abundance Factor (NSAF) [26,27]: for each protein, NSAF = (spectral count/protein length) divided by the sum of (spectral count/protein length) across all proteins in the same run, providing a between-run comparable abundance estimate.

Differentially expressed protein (DEP) criteria: A protein was reported as differentially expressed when (i) it was detected (NSAF > 0) in both the diabetic control and the contrasted treatment group; (ii) the spectral count was ≥5 in both groups; and (iii) the NSAF fold change versus DC was ≥1.2 (upregulated) or ≤0.83 (downregulated). Per-protein significance was assessed using the G-test (likelihood-ratio test, 1 df) on the 2 × 2 spectral-count contingency table—the standard significance test for label-free spectral counting [28]—and Benjamini–Hochberg false-discovery-rate (BH-FDR) correction was applied across all comparable proteins within each contrast (q < 0.05). The complete per-protein quantitative output (NSAF, fold change, log2 fold change, G statistic, *p* value, BH-adjusted q value, and pathway annotation) is provided as Supplementary Table S1.

Bioinformatics analysis: Functional annotation and pathway enrichment analyses of DEPs were conducted using the STRING database (version 12.0, https://string-db.org, accessed on 20 May 2025) for protein–protein interaction networks, gene ontology (GO) enrichment (biological processes, cellular components, molecular functions), and Kyoto Encyclopedia of Genes and Genomes (KEGG) pathway analysis. Enrichment was considered significant at FDR < 0.05.

### 2.12. Statistical Analysis

All statistical analyses were performed using SPSS software (version 26.0, IBM Corp., Armonk, NY, USA). Data are presented as mean ± standard deviation (SD). Normality of distribution was assessed using the Shapiro–Wilk test, and homogeneity of variances was evaluated using Levene’s test. All data met the assumptions of parametric testing. For comparisons among multiple experimental groups, one-way analysis of variance (ANOVA) was performed. When a significant overall effect was detected (*p* < 0.05), post hoc pairwise comparisons were conducted using Tukey’s honestly significant difference (HSD) test to control for family-wise error rate. For label-free proteomic data, per-protein significance was tested by the G-test (likelihood-ratio test, 1 df) on spectral counts [28], and multiple-comparison correction was applied using the Benjamini–Hochberg false-discovery-rate (FDR) method (q < 0.05). Replicates: For behavioural, metabolic, histological, and immunohistochemical analyses, biological replicates consisted of n = 8 independent animals per group. For label-free LC-MS/MS proteomics, individual animal lysates from each group were combined into a single representative pooled sample per group, and the resulting five group-level proteomes (NC, DC, PIO, S1, S2) were quantified by NSAF as detailed in Section 2.11. All in vitro assays (ELISA, immunohistochemistry quantification) were performed in triplicate as technical replicates, and the mean values were used for statistical analysis. Graphical representations were generated using GraphPad Prism (version 8.0.1, GraphPad Software, San Diego, CA, USA). Statistical significance was set at *p* < 0.05 for all comparisons unless otherwise specified.

### 2.13. Experimental Design and Timeline

**Figure 1:**
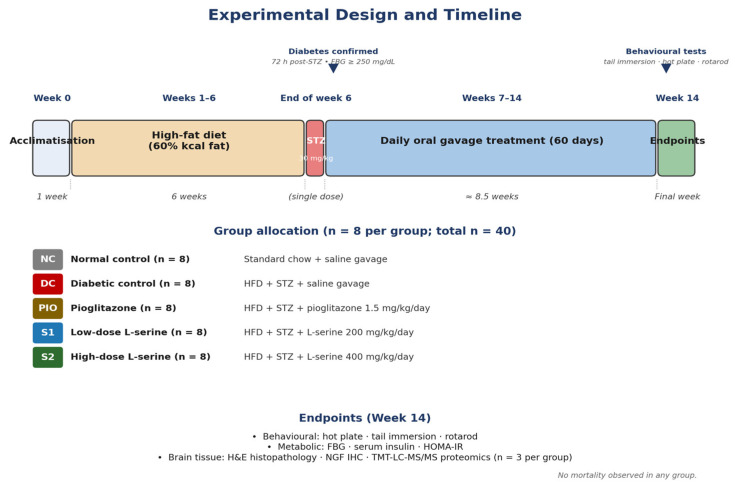
Schematic experimental design timeline. Male Wistar rats (n = 40) were acclimated for one week, then fed a high-fat diet (HFD, 60% fat) for 6 weeks. Diabetes was induced by a single intraperitoneal injection of streptozotocin (STZ, 30 mg/kg), confirmed 72 h later by fasting blood glucose ≥ 250 mg/dL. Rats were then allocated to five groups (n = 8 each): normal control (NC), diabetic control (DC), pioglitazone (PIO, 1.5 mg/kg/day), low-dose L-serine (S1, 200 mg/kg/day), and high-dose L-serine (S2, 400 mg/kg/day). All treatments were administered orally by gavage for 60 days. During week 14, behavioural tests (tail immersion, hot plate, rotarod) were performed, after which blood and brain tissues were collected for metabolic, histopathological, immunohistochemical, and proteomic analyses. No mortality occurred. Full details are provided in Section 2 (Materials and Methods). Abbreviations: HFD, high-fat diet; STZ, streptozotocin; FBG, fasting blood glucose; HOMA-IR, homeostatic model assessment of insulin resistance; NGF, nerve growth factor; LC-MS/MS, liquid chromatography-tandem mass spectrometry.

The complete experimental timeline spanned approximately 15 weeks, as outlined below. During week 0 (acclimatization), all animals were allowed a one-week habituation period to the animal facility. From weeks 1 through 6, rats assigned to diabetic groups were fed a high-fat diet (HFD, 60% kcal from fat), while the normal control group received standard chow. At the end of week 6, after an overnight fast, diabetes was induced by a single intraperitoneal injection of streptozotocin (STZ, 30 mg/kg). Seventy-two hours later (early week 7), fasting blood glucose was measured, and rats with glucose levels ≥ 250 mg/dL were considered diabetic and included in the study. Immediately after diabetes confirmation (week 7), the 60-day oral treatment period commenced, during which pioglitazone (1.5 mg/kg/day), low-dose L-serine (200 mg/kg/day), high-dose L-serine (400 mg/kg/day), or vehicle (sterile normal saline) was administered daily by gastric gavage. The treatment period extended from week 7 through week 14 (approximately 60 days). During the final week of treatment (week 14), behavioural assessments were performed in the following order: tail-immersion, hot-plate, and rotarod tests, with at least 24 h between tests to avoid fatigue or stress-related effects. At the end of week 14 (24 h after the last behavioural test), animals were euthanized. Blood was collected for metabolic assessments (fasting blood glucose, serum insulin, HOMA-IR), and brain tissues were harvested for histopathology (H&E staining), immunohistochemistry (NGF, synaptophysin, GFAP), and label-free LC-MS/MS proteomic analysis with NSAF quantification (Figure 1).

### 2.14. Ethics Statement

All animal procedures were conducted in accordance with the guidelines established by the Institutional Animal Care and Use Committee of Suez Canal University, Ismailia, Egypt, and followed the international standards outlined in the National Institutes of Health Guide for the Care and Use of Laboratory Animals (NIH Publication No. 85-23, revised 2011). The study protocol was approved by the Research Ethics Committee of the Faculty of Pharmacy, Suez Canal University, under approval code 202101MA5 (approved October 2021). All efforts were made to minimize animal suffering and to reduce the number of animals used in accordance with the 3R principles (Replacement, Reduction, Refinement).

## 3. Results

### 3.1. Thermal Latency Responses in Diabetic Neuropathy Rats Treated with L-Serine

**Figure 2:**
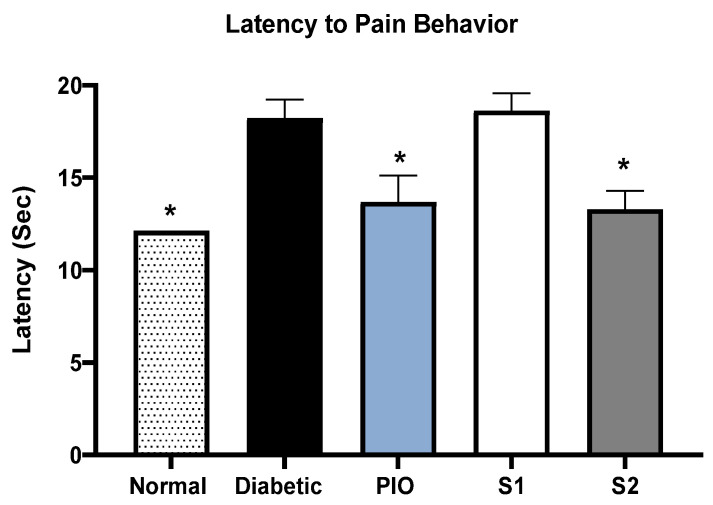
Effect of L-serine doses compared with pioglitazone (PIO) as a standard treatment on thermal pain sensitivity in diabetic rats assessed by the hot-plate test. Data are presented as mean ± SD (n = 8 per group). Statistical significance was determined using one-way ANOVA followed by Tukey’s post hoc test. * *p* < 0.05. Groups: PIO, pioglitazone (1.5 mg/kg/day); S1, L-serine (200 mg/kg/day); S2, L-serine (400 mg/kg/day).

Thermal nociception and pain sensitivity were evaluated using the hot-plate test, with thermal latency (time to withdrawal response) recorded in seconds (Figure 2). Normal control rats exhibited a baseline thermal latency of 12.5 ± 0.8 s. The diabetic control group showed a significant increase to 18.2 ± 1.2 s (*p* < 0.01 vs. normal control), indicating the reduced pain sensitivity characteristic of diabetic neuropathy. Pioglitazone (PIO) treatment reduced thermal latency to 14.1 ± 0.9 s (*p* < 0.01 vs. diabetic control), and high-dose L-serine (S2) similarly reduced it to 13.8 ± 0.7 s (*p* < 0.01 vs. diabetic control). In contrast, the low-dose L-serine treatment (S1) produced a thermal latency of 18.5 ± 1.1 s, which did not differ significantly from the diabetic control group (*p* > 0.05).

### 3.2. Evaluation of Thermal Sensitivity Using Tail-Immersion Test in Diabetic Neuropathy Rats Treated with L-Serine

**Figure 3:**
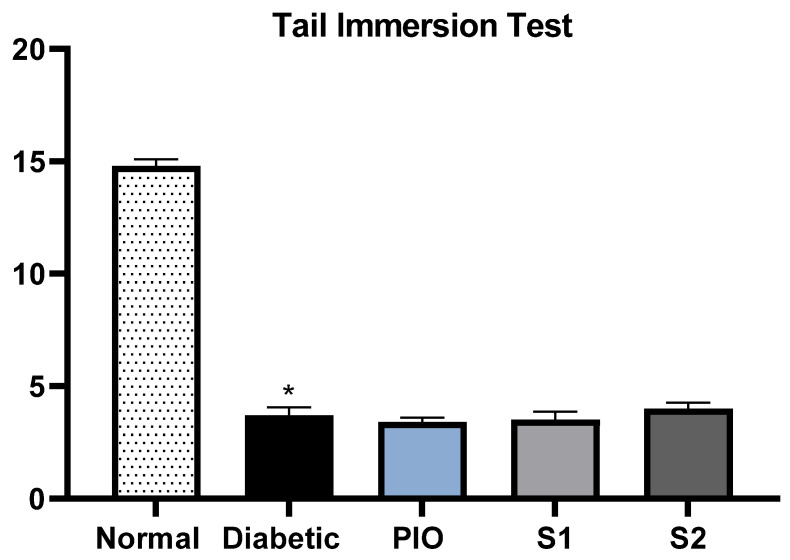
The impact of L-serine (at different doses) relative to the standard therapy pioglitazone (PIO) on thermal nociception in diabetic rats assessed by the tail-immersion test. Data are presented as mean ± SD (n = 8 per group). Statistical significance was determined using one-way ANOVA followed by Tukey’s post hoc test. * *p* < 0.05. Groups: PIO, pioglitazone (1.5 mg/kg/day); S1, L-serine (200 mg/kg/day); S2, L-serine (400 mg/kg/day).

The tail-immersion test was performed to further evaluate thermal sensitivity, recording the tail-withdrawal latency in seconds for each rat in each group (Figure 3). Normal control rats showed a baseline tail-withdrawal latency of 14.2 ± 0.9 s. The diabetic control group exhibited a significant reduction to 4.1 ± 0.6 s (*p* < 0.01 vs. normal control), confirming the thermal hypoalgesia characteristic of diabetic neuropathy. PIO treatment yielded a tail-withdrawal latency of 4.5 ± 0.4 s, which did not differ significantly from the diabetic control (*p* > 0.05). The low-dose and high-dose L-serine groups showed tail-withdrawal latencies of 4.2 ± 0.4 s and 4.8 ± 0.5 s, respectively; neither differed significantly from diabetic rats (*p* > 0.05).

### 3.3. Motor Coordination by Rotarod Test

**Figure 4:**
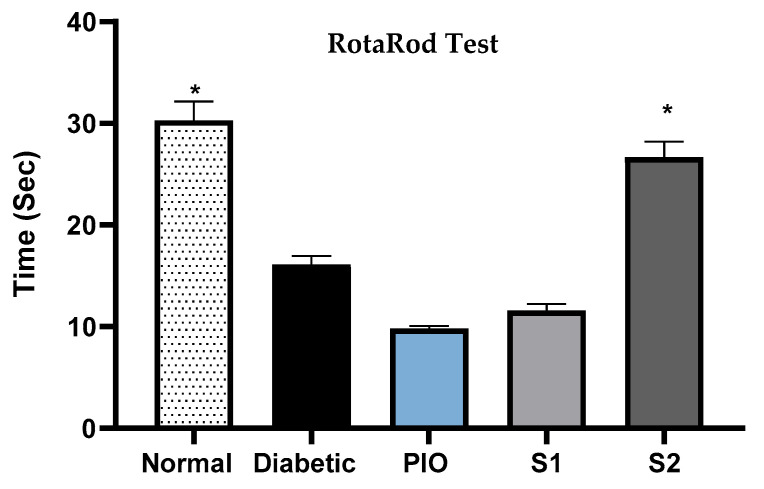
Comparing the effect of different L-serine doses against the standard drug pioglitazone (PIO) on motor coordination in diabetic rats assessed by the rotarod test. Data are presented as mean ± SD (n = 8 per group). Statistical significance was determined using one-way ANOVA followed by Tukey’s post hoc test. * *p* < 0.05. Groups: PIO, pioglitazone (1.5 mg/kg/day); S1, L-serine (200 mg/kg/day); S2, L-serine (400 mg/kg/day).

The rotarod test revealed significant differences in motor coordination among the experimental groups (Figure 4). Diabetic rats showed a significant reduction in time spent on the rotating rod, falling at 16.1 ± 0.85 s compared with 30.3 ± 1.85 s in the normal control group (*p* < 0.05), indicating motor impairment associated with diabetic neuropathy. PIO treatment led to a further reduction in motor performance, with the shortest latency to fall of any group at 9.8 ± 0.26 s (*p* < 0.05 vs. diabetic). In contrast, the high-dose L-serine (S2) group significantly improved motor performance to 26.67 ± 1.52 s (*p* < 0.05 vs. diabetic), whereas the low-dose (S1) group showed only a moderate, non-significant improvement (11.56 ± 0.64 s).

### 3.4. Comparative Impact of Low- and High-Dose L-Serine on Insulin Regulation in Diabetic Rats

**Figure 5:**
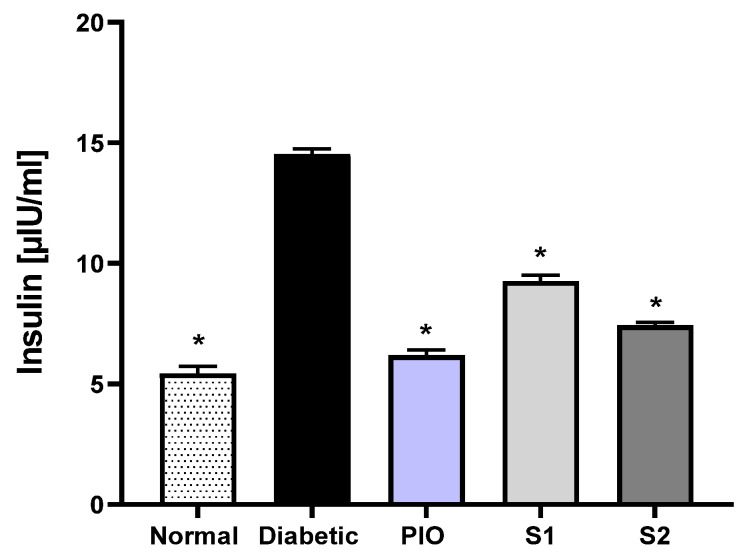
The impact of L-serine (at different doses) relative to the standard therapy pioglitazone (PIO) on serum insulin levels in diabetic rats. Data are presented as mean ± SD (n = 8 per group). Statistical significance was determined using one-way ANOVA followed by Tukey’s post hoc test. * *p* < 0.05. Groups: PIO, pioglitazone (1.5 mg/kg/day); S1, L-serine (200 mg/kg/day); S2, L-serine (400 mg/kg/day).

Serum insulin levels were significantly elevated in the diabetic group (14.53 ± 0.20 µIU/mL) compared with the normal control (5.43 ± 0.30 µIU/mL; *p* < 0.05), indicating a state of insulin resistance (Figure 5). Pioglitazone treatment produced a modest but significant reduction to 6.2 ± 0.20 µIU/mL (*p* < 0.05 vs. diabetic). Low-dose and high-dose L-serine significantly decreased insulin to 9.26 ± 0.25 and 7.43 ± 0.11 µIU/mL, respectively (*p* < 0.05 vs. diabetic), suggesting improved insulin sensitivity. Notably, the high-dose group showed a greater reduction than the low-dose group, although both remained above normal control levels.

### 3.5. Impact of L-Serine on Hyperglycemia in a Diabetic Rat Model

**Figure 6:**
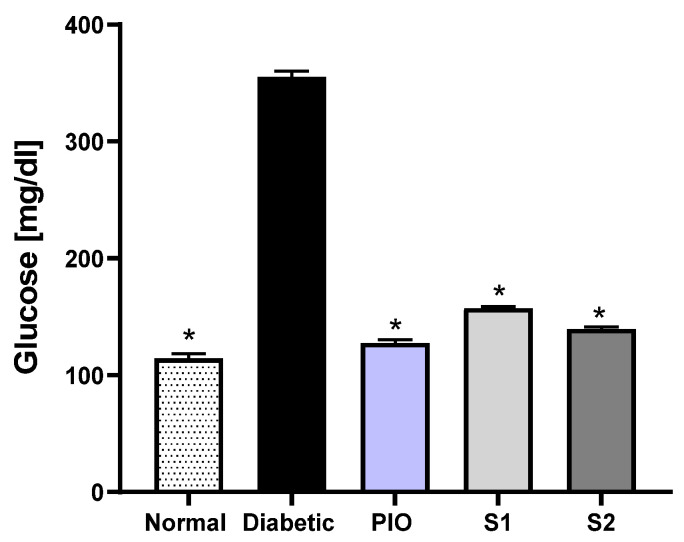
Effect of L-serine (S1, S2) and pioglitazone (PIO) on fasting blood glucose levels in diabetic rats. Data are presented as mean ± SD (n = 8 per group). Statistical significance was determined using one-way ANOVA followed by Tukey’s post hoc test. * *p* < 0.05. Groups: PIO, pioglitazone (1.5 mg/kg/day); S1, L-serine (200 mg/kg/day); S2, L-serine (400 mg/kg/day).

Diabetic rats exhibited a significant increase in fasting blood glucose to 355.33 ± 4.72 mg/dL compared with the normal control group (114.33 ± 4.04 mg/dL; *p* < 0.05), confirming successful induction of hyperglycemia (Figure 6). PIO treatment significantly reduced glucose levels to 127.67 ± 2.51 mg/dL (*p* < 0.05 vs. diabetic). Similarly, S1 and S2 treatment significantly reduced glucose to 157.33 ± 1.52 and 139.67 ± 1.52 mg/dL, respectively (*p* < 0.05 vs. diabetic), although both remained higher than in the normal group. No significant difference was observed between the low and high doses of L-serine, but both effectively ameliorated hyperglycemia.

### 3.6. Effect of L-Serine Treatment on Insulin Resistance in Diabetic Rats

**Figure 7:**
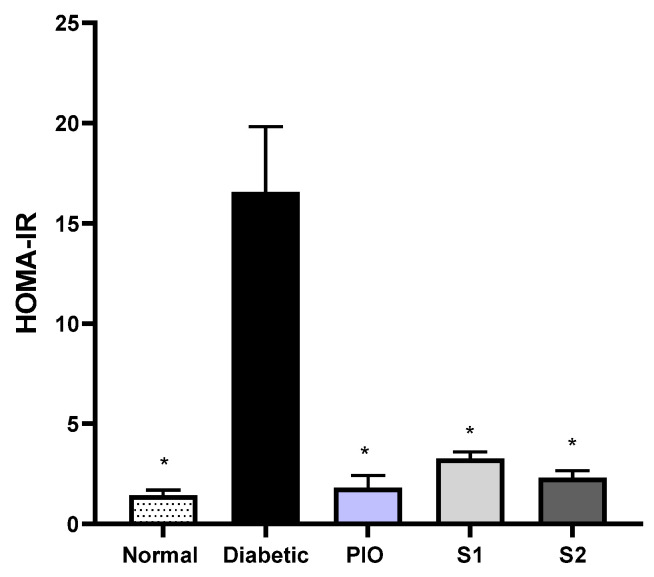
The effect of various L-serine doses in comparison to pioglitazone (PIO) used as the standard treatment on HOMA-IR values in diabetic rats. Data are presented as mean ± SD (n = 8 per group). Statistical significance was determined using one-way ANOVA followed by Tukey’s post hoc test. * *p* < 0.05. Groups: PIO, pioglitazone (1.5 mg/kg/day); S1, L-serine (200 mg/kg/day); S2, L-serine (400 mg/kg/day).

HOMA-IR (Homeostatic Model Assessment of Insulin Resistance) values were significantly elevated in the diabetic group (16.8 ± 3.2) compared with the normal control (1.2 ± 0.3), representing a 14-fold increase (*p* < 0.001) and confirming severe insulin resistance in the diabetic neuropathy model (Figure 7). Both low-dose (S1; 3.8 ± 0.5) and high-dose (S2; 2.1 ± 0.4) L-serine treatments significantly reduced HOMA-IR values compared with the diabetic group (*p* < 0.001), corresponding to reductions of 77.4% and 87.5%, respectively. The high-dose treatment achieved HOMA-IR values closer to normal control levels than the low-dose treatment. Notably, S2 showed superior efficacy compared with S1, although both groups remained significantly different from the normal control (*p* < 0.05).

### 3.7. Histopathological Evaluation of Brain Tissue in Diabetic Rats Treated with Pioglitazone and L-Serine

**Figure 8:**
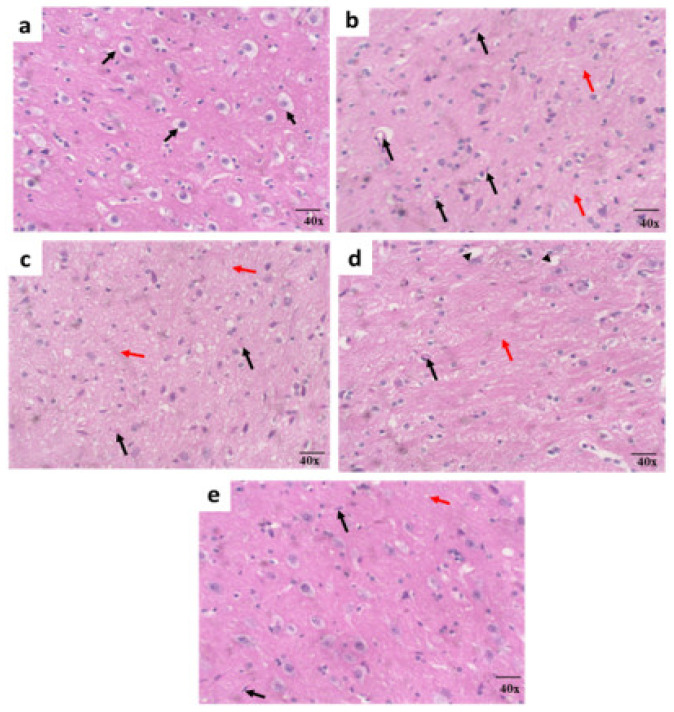
Histopathological examination of brain tissue in the experimental groups (**a**) normal rats, (**b**) diabetic rats, (**c**) pioglitazone-treated rats, (**d**) the S1 dose of L-serine, (**e**) the S2 dose of L-serine of brain tissues.

Histopathological analysis of brain sections revealed distinct alterations among the experimental groups (Figure 8). In the normal control group, no structural abnormalities were observed, and the neuronal architecture appeared intact without evidence of edema, red neurons, or gliosis. In contrast, the diabetic untreated group exhibited pronounced neuropathological changes, including marked perineuronal edema, degenerating neurons, and extensive gliosis, indicating severe neuronal injury and neuroinflammation. PIO treatment partially ameliorated these changes, with only a few red neurons and persistent gliosis remaining evident, suggesting moderate neuroprotection. The S1 group displayed mild perineuronal edema with a reduced number of red neurons and less gliosis, reflecting improvement over the diabetic group. The S2 group similarly showed fewer red neurons and less gliosis, although mild changes were still apparent, indicating a protective effect of L-serine against diabetes-induced neuropathological alterations. Collectively, these findings indicate that both PIO and L-serine exert neuroprotective effects in diabetic rats, with the S1 dose of L-serine showing notable attenuation of neuronal injury and edema.

### 3.8. Immunohistochemical Expression of Nerve Growth Factor (NGF) in Brain Tissue

**Figure 9:**
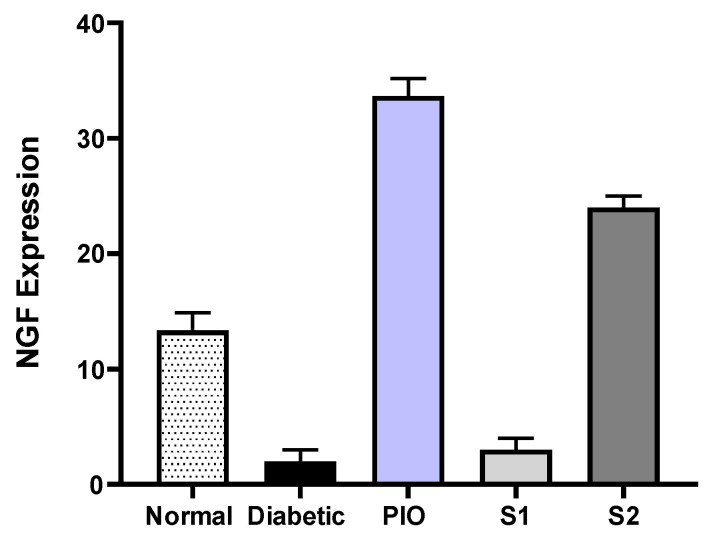
Semi-quantitative analysis of nerve growth factor (NGF) immunohistochemical expression in brain tissues of experimental groups. Data are presented as mean ± SD (n = 8 per group, 3 high-power fields per section). Statistical significance was determined using one-way ANOVA followed by Tukey’s post hoc test. Groups: PIO, pioglitazone (1.5 mg/kg/day); S1, L-serine (200 mg/kg/day); S2, L-serine (400 mg/kg/day).

**Figure 10:**
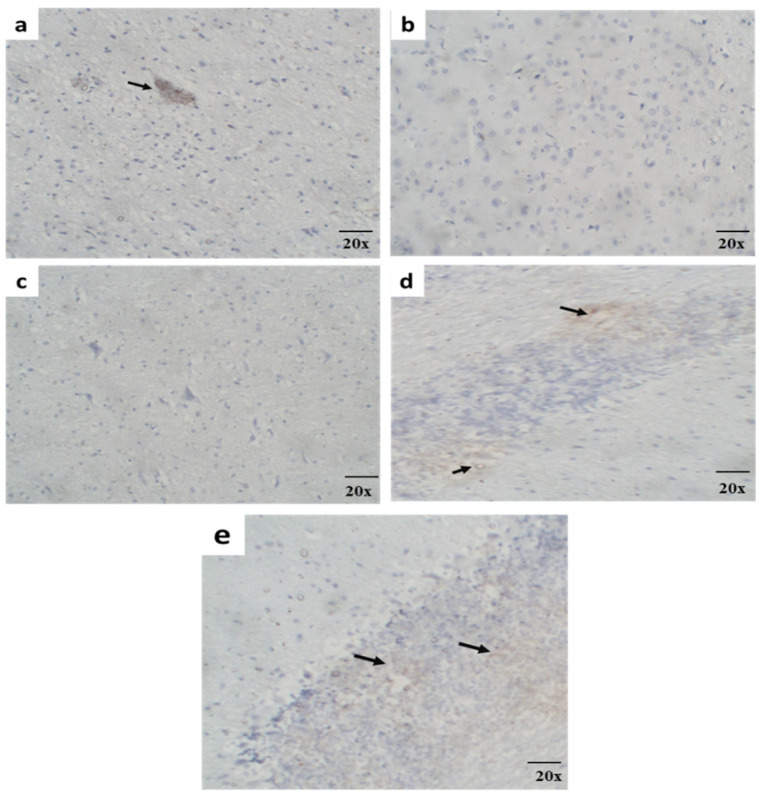
Representative photomicrographs of immunohistochemical staining for nerve growth factor (NGF) in brain tissue of the experimental groups: (**a**) normal rats, (**b**) diabetic rats, (**c**) pioglitazone-treated rats, (**d**) the S1 dose of L-serine, and (**e**) the S2 dose of L-serine. Arrows indicate positive NGF immunoreactivity (brown staining); original magnification ×20. Groups: PIO, pioglitazone (1.5 mg/kg/day); S1, L-serine (200 mg/kg/day); S2, L-serine (400 mg/kg/day).

Immunohistochemical staining showed a faint positive reaction for NGF in brain sections of the diabetic control group, in contrast to the markedly stronger NGF immunoreactivity observed in the normal control (Figure 9 and Figure 10). PIO treatment produced a marked restoration of NGF expression, reaching the highest levels among all experimental groups (34.5 ± 2.3). This represented a 12-fold increase compared to the diabetic group and a 2.6-fold increase compared to the normal control group (*p* < 0.001). The S1 dose of L-serine treatment group showed a modest but significant improvement in NGF expression (4.2 ± 0.8) compared to the diabetic group, though this increase was relatively small (50% improvement, *p* < 0.05). The S2 dose of L-serine showed substantially greater efficacy, with NGF expression reaching 24.1 ± 1.8, representing an 8.6-fold increase compared to the diabetic group (*p* < 0.001) and a 5.7-fold increase compared to the S1 dose group (*p* < 0.001). However, the S2 dose of L-serine group showed significantly lower NGF expression compared to the PIO monotherapy group (*p* < 0.01). Immunohistochemical analysis revealed distinct patterns of NGF expression across different brain regions (Figure 10). In the normal control group, NGF immunoreactivity was primarily localized in neuronal cell bodies and dendrites, with moderate staining intensity in the hippocampus, cortex, and brainstem regions. The diabetic group showed severely diminished NGF staining, with sparse immunoreactivity limited to scattered neurons and reduced dendritic labelling. PIO treatment restored robust NGF expression throughout the brain parenchyma, with intense immunoreactivity observed in pyramidal neurons of the hippocampus, cortical layers II–V, and brainstem nuclei. The staining pattern suggested enhanced NGF synthesis and transport, with prominent labelling of neuronal processes and synaptic terminals. L-serine treatment groups showed variable restoration patterns, with the S2 dose group demonstrating more widespread NGF immunoreactivity compared to the S1 dose treatment. The S2 dose L-serine therapy appeared to preferentially enhance NGF expression in specific neuronal populations, particularly in regions associated with memory formation and synaptic plasticity.

### 3.9. Proteomics Analysis of Low-Dose L-Serine Treatment in Diabetic Neuropathy in Brain Tissue

**Figure 11:**
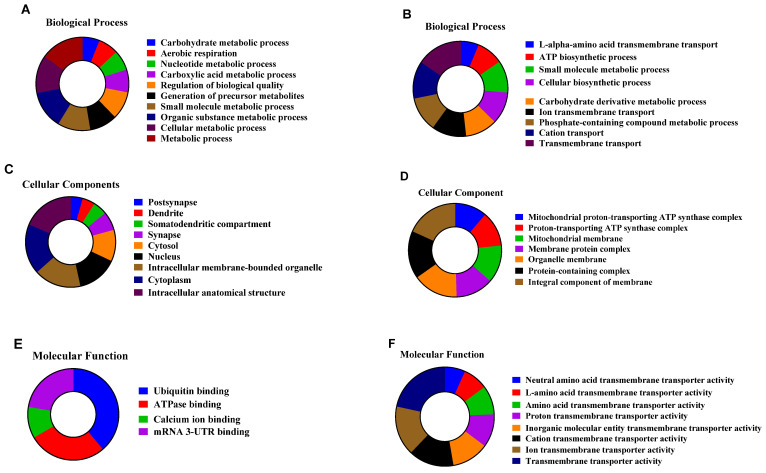
Functional enrichment analysis of differentially expressed brain proteins in diabetic neuropathy rats following low dose (S1) of L-serine treatment. The left panels (**A**,**C**,**E**) represent upregulated proteins, while the right panels (**B**,**D**,**F**) represent downregulated proteins. (**A**,**B**) Biological processes enriched in differentially expressed proteins. (**C**,**D**) Cellular component distribution showing protein localization. (**E**,**F**) Molecular function enrichment highlighting regulatory roles. Colours represent distinct functional categories as indicated in the legends. Data represents proteins with significant differential expressions (*p* < 0.05, fold change ≥ 1.2 or ≤0.83) following low-dose L-serine treatment compared to diabetic control group.

Figure 11 presents functional enrichment analysis of differentially expressed brain proteins following low-dose L-serine (S1, 200 mg/kg/day) treatment compared to diabetic controls. These comparisons derive from a single pooled sample per group and are therefore exploratory and hypothesis-generating; the enrichment patterns described below identify candidate processes for future validation rather than established mechanisms. Upregulated biological processes (Figure 11A) were dominated by carbohydrate metabolic process (6.14% and 7.59%), amino acid metabolic process (7.02%), nucleotide metabolic process (7.02%), and small molecule metabolic process (11.40%), supporting glucose utilization, energy production, and cellular repair. Downregulated biological processes (Figure 11B) included ATP biosynthetic process (9.09%) and transmembrane transport (15.45%), suggesting a protective reduction in energy demand and glucose influx. Upregulated cellular components (Figure 11C) included cytoplasm (17.98%), synapse (6.74%), and dendrite (4.49%), indicating enhanced metabolic activity and synaptic integrity. Downregulated cellular components (Figure 11D) included mitochondrial ATP synthase complex (11.30%) and mitochondrial membrane (13.04%), likely representing a protective adaptation against oxidative stress. Upregulated molecular functions (Figure 11E) showed enrichment in ubiquitin binding (38.80%), ATPase binding (27.78%), and calcium ion binding (11.11%), which are associated with protein quality control, energy-dependent processes, and neurotransmitter release, and may be relevant to the metabolic and behavioural changes observed; this single pooled comparison cannot, however, establish causation. Downregulated molecular functions (Figure 11F) were dominated by transmembrane transport activity (21.62%), indicating reduced glucose and ion influx that may lower oxidative stress.

In summary, low-dose L-serine was associated with enrichment of carbohydrate and amino acid metabolism, with reduced glucose-influx and energy-demand pathways, and with preserved synaptic-component representation. Enrichment of ubiquitin binding (38.80%) and ATPase binding (27.78%), together with reduced representation of mitochondrial complexes and transmembrane transport, constitutes an exploratory pattern that is consistent with—but does not by itself establish—improved insulin sensitivity and neuroprotection.

### 3.10. Proteomics Analysis of High-Dose L-Serine Treatment in Diabetic Neuropathy in Brain Tissue

**Figure 12:**
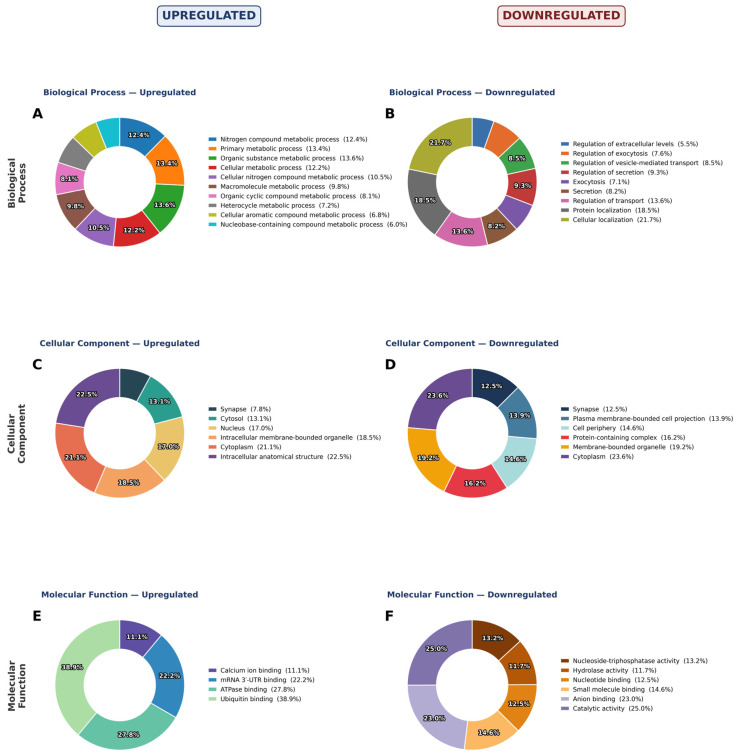
Functional enrichment categorization of differentially expressed proteins in brain tissue following high dose (S2) of L-serine treatment in diabetic neuropathy rat model. The pie charts showing the distribution of left panels (**A**,**C**,**E**) represent upregulated proteins, while the right panels (**B**,**D**,**F**) represent downregulated proteins classified by gene ontology terms. (**A**,**B**) Biological process enrichment showing shifts in metabolic, catabolic, and regulatory pathways. (**C**,**D**) Cellular component distribution highlighting synaptic, cytosolic, and organelle-related changes. (**E**,**F**) Molecular function enrichment indicating enhanced proteostasis, ATPase and calcium binding (upregulation) and reduced nucleotide/ribonucleotide binding and hydrolase activity (downregulation). Colours represent distinct functional categories as indicated in the legends. Data represents proteins with significant differential expressions (*p* < 0.05, fold change ≥ 1.2 or ≤0.83) following L-serine treatment compared to diabetic control group.

Figure 12 presents functional enrichment analysis of differentially expressed brain proteins following high-dose L-serine (S2, 400 mg/kg/day) treatment compared to diabetic controls. Upregulated biological processes (Figure 12A) included nitrogen compound metabolic process (12.37%), primary metabolic process (13.40%), and organic substance metabolic process (13.60%), indicating enhanced amino acid and carbohydrate metabolism. Downregulated biological processes (Figure 12B) included regulation of extracellular level (5.45%), regulation of exocytosis (7.61%), protein localization (18.48%), and cellular localization (21.74%), suggesting reduced inflammatory and stress responses. Upregulated cellular components (Figure 12C) included synapse (7.84%), cytosol (13.07%), and nucleus (16.99%), indicating enhanced sites for neuronal signalling and protein synthesis. Downregulated cellular components (Figure 12D) included synapse (12.50%), plasma membrane-bounded cell projection (13.89%), and cell periphery (14.58%), which may represent protective pruning of excessive synaptic connections under hyperglycemia. Upregulated molecular functions (Figure 12E) showed enrichment in ubiquitin binding (38.89%), ATPase binding (27.78%), mRNA 3′-UTR binding (22.22%), and calcium ion binding (11.11%). Ubiquitin binding enhances clearance of damaged proteins, ATPase binding supports energy production, and calcium ion binding may relate to neurotransmitter release—exploratory associations that may be relevant to neuroprotection and behavioural recovery—although the pooled discovery design precludes a direct mechanistic claim. Downregulated molecular functions (Figure 12F) included nucleotide-triphosphate activity (13.25%), hydrolase activity (11.66%), and catalytic activity (25.04%), representing a protective reduction in excessive metabolic reactions under hyperglycemia.

In summary, high-dose L-serine enhanced nitrogen and energy metabolism, reduced inflammatory signalling, and preserved synaptic integrity. The enrichment of ubiquitin, ATPase, and calcium binding is consistent with protein quality control, energy production, and neurotransmission—exploratory patterns that may relate to, but do not by themselves demonstrate, neuroprotection and behavioural recovery.

### 3.11. Proteomic Analysis of Pioglitazone Treatment in Diabetic Neuropathy Brain Tissue

**Figure 13:**
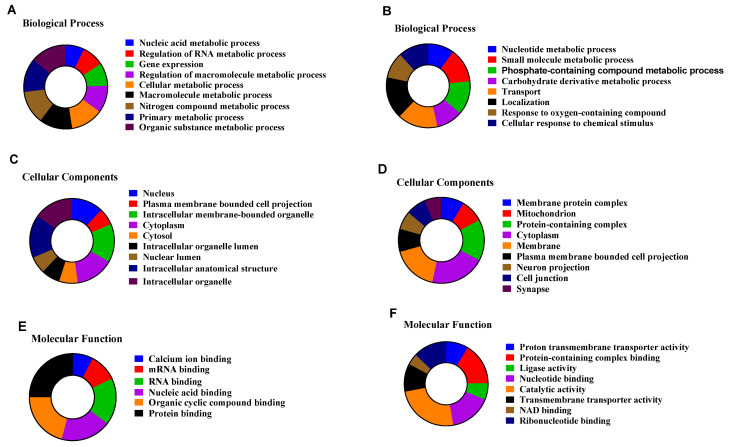
Functional enrichment categorization of differentially expressed proteins in brain tissue following pioglitazone treatment in diabetic neuropathy rat model. The pie charts showing the distribution of left panels (**A**,**C**,**E**) represent upregulated proteins, while the right panels (**B**,**D**,**F**) represent downregulated proteins classified by gene ontology terms. (**A**,**B**) Biological processes demonstrate upregulation of nucleic acid metabolic processes, DNA metabolism, and macromolecule metabolism versus downregulation of nucleotide metabolism and transport processes. (**C**,**D**) Cellular components reveal enhanced nucleus-associated proteins, and organellar structures contrasted with suppressed membrane protein complexes, and mitochondrial stress components. (**E**,**F**) Molecular functions show increased catalytic activity, and binding functions alongside decreased transmembrane transporter activity, and pathological protein interactions. Colors represent distinct functional categories as indicated in the legends. Data represents proteins with significant differential expressions (*p* < 0.05, fold change ≥ 1.2 or ≤0.83) following PIO treatment compared to diabetic control group.

Figure 13 presents a functional enrichment analysis of differentially expressed brain proteins following pioglitazone (1.5 mg/kg/day) treatment compared to diabetic controls. Biological Processes (Figure 13A,B): upregulated biological processes (Figure 13A) were dominated by nucleic acid metabolic process (7.21%), regulation of RNA metabolic process (8.52%), gene expression (8.88%), regulation of macromolecular metabolic process (10.80%), and primary metabolic process (13.11%). The enrichment of nucleic acid-related pathways is consistent with pioglitazone’s known mechanism as a PPAR-γ agonist, which exerts its effects primarily through transcriptional regulation. This signature indicates enhanced gene expression and RNA processing in diabetic brain tissue following pioglitazone treatment. Downregulated biological processes (Figure 13B) included nucleic acid metabolic process (10.08%), small molecule metabolic process (13.18%), phosphorus-containing compound metabolic process (13.18%), carbohydrate derivative metabolic process (10.08%), transport (15.60%), and cellular response to chemical stimuli (11.63%). The downregulation of these pathways may represent a normalization of aberrantly activated metabolic and stress-response pathways in the diabetic state, rather than a detrimental effect. 

Cellular Components (Figure 13C,D): Upregulated cellular components (Figure 13C) included nucleus (12.14%), intracellular anatomical structure (16.43%), intracellular organelle (15.00%), and nuclear lumen (6.43%). This distribution is consistent with pioglitazone’s primary site of action—the nucleus—where PPAR-γ binds to DNA response elements and regulates target gene transcription. The enrichment of nuclear and organellar components supports the interpretation that pioglitazone acts through transcriptional reprogramming. Downregulated cellular components (Figure 13D) included membrane protein complex (3.62%), mitochondrion (3.62%), protein-containing complex (15.52%), membrane (17.24%), and cell junction (7.47%). The downregulation of membrane-associated and mitochondrial components may reflect reduced metabolic and inflammatory stress in the diabetic brain, as chronic hyperglycemia typically upregulates these compartments pathologically. Molecular Functions (Figure 13E,F): upregulated molecular functions (Figure 13E) showed enrichment in protein binding (25.00%), organic cyclic compound binding (20.83%), nucleic acid binding (18.75%), RNA binding (17.36%), and mRNA binding (10.42%). These functions support pioglitazone’s role in regulating gene expression, RNA processing, and protein–protein interactions essential for cellular repair and metabolic adaptation. Downregulated molecular functions (Figure 13F) included protein-containing complex binding (16.13%), catalytic activity (24.73%), transmembrane transporter activity (10.50%), and nucleotide binding (10.13%). The downregulation of catalytic and transporter activities may represent a protective reduction in excessive metabolic flux and ion transport under hyperglycemic conditions.

In summary, pioglitazone (1.5 mg/kg/day) induced a proteomic signature characterized by the upregulation of nucleic acid metabolic processes (7.21%), regulation of RNA metabolism (8.52%), and gene expression (8.88%), consistent with its PPAR-γ-mediated transcriptional mechanism. Analysis of cellular components revealed enrichment of nuclear (12.14%) and organellar compartments, consistent with the nucleus as the primary site of action. Downregulated pathways included small molecule metabolism, transport, and membrane-associated components, suggesting a normalization of diabetes-induced metabolic and inflammatory stress. At the molecular level, upregulated protein binding (25.00%) and nucleic acid binding (18.75%) support pioglitazone’s role in transcriptional regulation, while downregulated catalytic (24.73%) and transporter activities (10.50%) indicate reduced metabolic burden. These proteomic changes, however, did not translate into behavioural recovery in the present study, possibly due to species-specific differences in PPAR-γ signalling or the suprapharmacological dose used.

## 4. Discussion

The data reported here are consistent with dose-dependent metabolic, neurobehavioural, histopathological, and proteomic effects of L-serine in a rat model of diabetic neuropathy. High-dose L-serine produced the strongest HOMA-IR reduction (87.5%), while the low dose also showed significant improvement (77.4%). Both doses ameliorated neuronal degeneration, perineuronal edema, and gliosis. Proteomics revealed distinct dose-specific mechanisms: low-dose treatment predominantly upregulated metabolic and biosynthetic pathways, whereas high-dose treatment enhanced synaptic, neurotransmitter-regulatory, and proteostasis-related processes. Pioglitazone showed strong effects on nucleic acid metabolism and DNA repair pathways but provided less behavioural recovery. Together, these findings are consistent with the hypothesis that L-serine warrants further preclinical evaluation as a candidate adjunctive agent in diabetic neuropathy, pending the orthogonal-validation and disease-mechanistic experiments outlined in the Section Limitations of the Study. We note at the outset that the molecular and proteomic findings reported below derive from cerebral tissue rather than from peripheral nerves, and therefore inform the central component of the diabetic-neuropathy phenotype rather than directly establishing mechanisms operating at the peripheral nerve or dorsal root ganglion; this inferential boundary is reflected throughout the Discussion and explicitly enumerated in the Section Limitations of the Study.

Metabolic dysfunction is a central driver of DN progression [4,14]. Consistent with this, diabetic rats developed severe hyperglycemia, hyperinsulinemia, and a 14-fold elevation in HOMA-IR. L-serine supplementation produced substantial metabolic improvement, with both doses yielding significant reductions in glucose, insulin, and insulin resistance. This aligns with evidence that L-serine enhances mitochondrial function, reduces oxidative stress, and supports insulin secretion and sensitivity [18,19,20]. The more pronounced metabolic response at the low dose suggests a therapeutic window in which L-serine supports energy metabolism without overburdening serine-dependent pathways such as sphingolipid synthesis.

Recent mechanistic work demonstrates that impaired serine availability drives accumulation of neurotoxic deoxysphingolipids that exacerbate insulin resistance and neuropathy [22]. By restoring serine-dependent pathways, L-serine likely reduces these toxic lipids, improving insulin receptor signalling and metabolic flexibility. The metabolic findings also align with 2025 reports indicating that amino acid-based metabolic reprogramming represents a viable strategy for mitigating DN-associated metabolic memory [3,6,24].

Diabetic rats exhibited impaired nociception and motor coordination—hallmarks of sensory and sensorimotor neuropathy [7,8]. High-dose L-serine significantly restored thermal sensitivity and improved rotarod performance, whereas the low dose produced only partial effects. The divergence between metabolic and behavioral endpoints indicates that behavioural improvement may depend more on neurotrophic and synaptic mechanisms than on metabolic normalization alone.

The contrast between hot-plate and tail-immersion responses provided additional insight. Improvements were more pronounced in supraspinally mediated responses (hot plate), suggesting that L-serine preferentially restores higher-order pain processing pathways. This is consistent with emerging evidence that central neuronal circuits—including hippocampal and cortical regions—are affected in DN and contribute to altered pain perception and cognitive symptoms [9,25].

Two further points warrant explicit comment. First, the directional divergence between the hot-plate (latency increased in diabetic controls) and tail-immersion (latency decreased in diabetic controls) responses is not a contradiction but reflects the distinct neural substrates of these two assays: hot-plate responding integrates supraspinal pain processing, whereas tail-flick is a predominantly spinal reflex. The two endpoints can therefore move in opposite directions in early diabetic neuropathy, and L-serine’s preferential restoration of the supraspinal response is consistent with its central, NMDA- and synaptic-pathway-related mechanisms. Second, L-serine did not improve every behavioural endpoint examined: tail-immersion latency was not significantly restored in either dose group, motor coordination improved only at the high dose, and metabolic correction was nearly equivalent across doses. We therefore avoid any inference of broad neuropathic-function rescue and frame the behavioural improvements as endpoint-specific. Definitive characterization will require disease-mechanistic measures that were not performed here, including nerve-conduction velocity, von Frey mechanical-threshold testing, and intraepidermal nerve-fibre density quantification.

Motor recovery with L-serine reflects its role in phosphatidylserine and D-serine synthesis, both of which modulate neuronal membrane stability and NMDA receptor-dependent plasticity [21]. These findings integrate well with recent data showing that serine-targeted interventions enhance sensorimotor integration in diabetic rodents [20,22].

Proteomic profiling identified patterns that paralleled the functional outcomes; because these derive from pooled, discovery-level data, they are interpreted as exploratory associations rather than established mechanisms. Low-dose L-serine upregulated proteins involved in carbohydrate and lipid metabolism, biosynthesis, and small-molecule processing, a pattern consistent with metabolic reprogramming that may support neuronal resilience. The downregulation of mitochondrial ATP synthase complexes may reflect a protective shift toward reduced oxidative stress under hyperglycemic conditions.

High-dose L-serine was associated with more pronounced changes in synaptic- and neurotrophic-related proteins, including altered representation of proteins linked to neurotransmitter regulation, cytokine-mediated signalling, synaptic components, and membrane-bound projections. These changes are consistent with L-serine’s established role in sphingolipid biosynthesis and synaptic membrane maintenance, as well as its contribution to NMDA receptor co-agonist production through D-serine [21].

Recent 2025 proteomic studies of DN report that the restoration of synaptic proteins and proteostasis networks correlates with neuropathic recovery [25], consistent with the present exploratory observations. The enrichment of nucleotide-binding, hydrolase, and proteasomal activities in our dataset is consistent with enhanced protein turnover and reduced proteotoxic stress—processes critical for neuronal survival under diabetic conditions. NGF immunohistochemistry was concordant with the proteomic signatures: high-dose L-serine was associated with an 8.6-fold higher NGF immunoreactivity than diabetic controls, which may reflect enhanced neurotrophin support, but was not corroborated by an orthogonal assay in the present study. This aligns with studies showing that serine-driven lipid remodelling modulates neurotrophic pathways and supports axonal repair [22].

Across all metabolic endpoints—fasting blood glucose, serum insulin, and HOMA-IR—the high dose produced numerically and statistically superior correction relative to the low dose, while the low dose itself produced substantial metabolic benefit. On the behavioural and neurotrophic endpoints, however, only the high dose meaningfully restored thermal sensitivity, motor coordination, and NGF expression; the low dose produced minimal behavioural change. Taken together, these data are consistent with a partly saturating metabolic effect—where additional serine yields modest further metabolic gain beyond the low dose—coupled with a clearly dose-dependent behavioural and synaptic response that emerges only at the higher exposure. We frame this dose-dependent pattern as a working hypothesis rather than an established mechanism: it is derived from a two-dose preclinical study, it has not been corroborated by orthogonal molecular validation, and formal multi-dose–response studies are required before any mechanistic claim can be advanced.

This dose-dependent divergence suggests that L-serine engages distinct molecular pathways depending on its availability. Excess serine may shift metabolism toward sphingolipid and phosphatidylserine synthesis, enhancing synaptic function but providing diminishing metabolic returns. These findings parallel 2024–2025 reports of dose-specific amino acid effects in diabetes-related neurodegeneration [22,23,24,25,30,31,32,33,34,35,36].

Pioglitazone improved glycemic control and partially ameliorated neuronal injury, consistent with its known anti-inflammatory and insulin-sensitizing properties [12]. However, its behavioural improvements were less pronounced than those of high-dose L-serine, and motor performance was unexpectedly worsened. Proteomics revealed that pioglitazone predominantly enhanced nucleic acid metabolism, DNA repair, and macromolecule metabolism—findings consistent with the drug’s PPAR-γ-mediated transcriptional reprogramming. The NGF recovery observed under pioglitazone may reflect the modulation of neurotrophin expression via anti-inflammatory mechanisms, a pattern consistent with recent clinical observations [14]. Nevertheless, L-serine provided broader synaptic and proteostasis benefits, suggesting complementary mechanisms.

Whether combining L-serine with pioglitazone—or other PPAR-γ agonists—would yield additive or synergistic benefit cannot be addressed by the present data and remains a speculative possibility for future, appropriately designed studies. Any such combination would need to be tested directly before therapeutic inferences are drawn.

### Limitations of the Study

Several limitations should be acknowledged. First, the detailed molecular, histological, immunohistochemical, and proteomic analyses were performed on brain tissue rather than on peripheral nerve or dorsal root ganglia. While peripheral neuropathy was functionally confirmed by validated behavioural tests, and although central mechanisms are increasingly recognized as contributors to neuropathic pain, follow-up studies should prioritize parallel analysis of sciatic nerve and dorsal root ganglia to directly correlate central findings with peripheral pathology. Second, the high-fat-diet/low-dose-streptozotocin rat model does not fully recapitulate the multifactorial pathophysiology of human type 2 diabetic neuropathy. Third, only two doses of L-serine and a single comparator dose of pioglitazone were tested; formal dose–response studies for both compounds are required to establish optimal dosing. Fourth, the translational relevance of these findings to human diabetic neuropathy remains unknown and requires clinical investigation. Fifth, the proteomic analysis was discovery-based and hypothesis-generating; the identified pathways require independent validation by orthogonal techniques such as Western blot, quantitative PCR, or targeted mass-spectrometric assays. Finally, the use of a single-sex (male) cohort limits generalisability, and sex-stratified replication is needed. Confirmation of these results and their translation to human diabetic neuropathy will require independent replication, sex-stratified replication, dose–response studies, orthogonal molecular validation, and ultimately, clinical trials. We additionally acknowledge three further constraints raised during peer review: (i) whole-brain proteomic profiling, while informative for central neurometabolic remodelling, cannot by itself establish the molecular mechanisms of diabetic peripheral neuropathy and our findings on this point should be read as hypothesis-generating; (ii) the disease-mechanistic neurophysiological assays of choice—nerve-conduction velocity, von Frey mechanical thresholds, and intraepidermal nerve-fibre density—were not performed in this discovery study and are a high-priority component of the planned follow-up work; and (iii) the NGF immunohistochemical findings were not corroborated by an orthogonal modality (Western blot, ELISA, qPCR, or immunofluorescence) within this study, and orthogonal validation of NGF expression and of the top differentially expressed proteomic targets is required before any mechanistic claim can be advanced. In response to peer-review feedback, we are explicit on three further points. First, the disease-mechanistic neurophysiological assays—nerve-conduction velocity, von Frey mechanical-threshold testing, and intraepidermal nerve-fibre density quantification—are the assays best able to confirm peripheral neuropathy at the structural and electrophysiological level; these were not performed in the discovery study reported here, and we identify them as the first deliverables of the planned follow-up programme. Second, orthogonal validation of NGF expression by Western blot, ELISA, and qPCR is similarly identified as a near-term priority. Third, we re-emphasize that whole-brain proteomic profiling, however informative for central neurometabolic remodelling, cannot by itself establish mechanisms operating at the peripheral nerve or dorsal root ganglion; the inferences drawn in this manuscript are accordingly framed as hypothesis-generating in every section.

## 5. Conclusions

In this exploratory preclinical study, L-serine supplementation was associated with measurable metabolic and behavioural improvements, together with reduced central neuronal injury, in a high-fat-diet/low-dose-streptozotocin rat model exhibiting a diabetic-neuropathy phenotype. Diabetic animals exhibited hyperglycaemia, hyperinsulinaemia, motor dysfunction, reduced pain thresholds, and pronounced brain histopathological alterations. Pioglitazone partially attenuated these outcomes, whereas L-serine improved glucose and insulin homeostasis, improved motor coordination and thermal pain thresholds, and attenuated neuronal injury in brain tissue. The proteomic data generate the testable hypothesis of dose-dependent effects: at lower doses L-serine appears to favour metabolic and biosynthetic remodelling, whereas at higher doses it favours synaptic, neurotransmission-related, and proteostatic pathways. Pioglitazone, by contrast, predominantly engaged transcriptional regulation and cellular-repair pathways. Taken together, these findings provide an exploratory, hypothesis-generating signal for L-serine in experimental diabetic neuropathy; they do not establish a therapeutic mechanism, and the central proteomic findings in particular require orthogonal validation. Any relevance to human diabetic neuropathy is at present remote and speculative, and would require formal dose–response studies, direct peripheral-nerve assessment, orthogonal molecular validation, and ultimately controlled clinical investigation.

## Data Availability

The original contributions presented in this study are included in the article. Further inquiries can be directed to the corresponding author.

## Supplementary Materials

The following supporting information can be downloaded at: https://www.mdpi.com/article/10.3390/biom16060881/s1, Table S1: Differentially expressed brain proteins in HFD/STZ-induced diabetic rats following pioglitazone, low-dose, or high-dose L-serine treatment (label-free spectral-counting quantification).
